# Water Kefir and Derived Pasteurized Beverages Modulate Gut Microbiota, Intestinal Permeability and Cytokine Production In Vitro

**DOI:** 10.3390/nu13113897

**Published:** 2021-10-29

**Authors:** Marta Calatayud, Rosa Aragao Börner, Jonas Ghyselinck, Lynn Verstrepen, Jelle De Medts, Pieter Van den Abbeele, Claire L. Boulangé, Sarah Priour, Massimo Marzorati, Sami Damak

**Affiliations:** 1 ProDigest, Technologiepark 82, 9052 Zwijnaarde, Belgium; Marta.Calatayud@prodigest.eu (M.C.); Jonas.Ghyselinck@prodigest.eu (J.G.); Lynn.Verstrepen@prodigest.eu (L.V.); Jelle.DeMedts@prodigest.eu (J.D.M.); Pieter.VandenAbbeele@telenet.be (P.V.d.A.); 2 Center for Microbial Ecology and Technology (CMET), Faculty of Bioscience Engineering, Ghent University, Coupure Links 653, 9000 Ghent, Belgium; 3 Nestlé Institute of Health Sciences, and Nestlé Institute of Material Sciences, Nestlé Research, Société des Produits Nestlé S.A., Vers-chez-les-Blanc, 1000 Lausanne, Switzerland; claire.boulange@rd.nestle.com (C.L.B.); Sarah.Priour@rd.nestle.com (S.P.); sami.damak@rdls.nestle.com (S.D.)

**Keywords:** water kefir, microbiota, immunomodulation, short-chain fatty acids, gut barrier

## Abstract

Fermentation is an ancient food preservation process, and fermented products have been traditionally consumed in different cultures worldwide over the years. The interplay between human gut microbiota, diet and host health is widely recognized. Diet is one of the main factors modulating gut microbiota potentially with beneficial effects on human health. Fermented dairy products have received much attention, but other sources of probiotic delivery through food received far less attention. In this research, a combination of in vitro tools mimicking colonic fermentation and the intestinal epithelium have been applied to study the effect of different pasteurized and non-pasteurized water kefir products on gut microbiota, epithelial barrier function and immunomodulation. Water kefir increased beneficial short-chain fatty acid production at the microbial level, reduced detrimental proteolytic fermentation compounds and increased *Bifidobacterium* genus abundance. The observed benefits are enhanced by pasteurization. Pasteurized products also had a significant effect at the host level, improving inflammation-induced intestinal epithelial barrier disruption and increasing IL-10 and IL-1β compared to the control condition. Our data support the potential health benefits of water kefir and demonstrate that pasteurization, performed to prolong shelf life and stability of the product, also enhanced these benefits.

## 1. Introduction

Gut microbes play a key role in human health, including the development of the immune system [1,2,3] and host metabolism [4,5]. In addition, several studies have shown associations between changes in the gut microbiota and various diseases including allergy [6], inflammatory bowel disease [7], diabetes [8,9], asthma [10,11], and obesity [12].

Fermented products have been linked to health benefits, potentially through gut microbiota modulation. Indeed, fermented products can be considered a source of probiotic microorganisms, defined as live microorganisms that confer a health benefit on the host [13].

Among fermented food products, water kefir, is made by fermentation of simple carbohydrate solutions (e.g., sucrose, glucose, fructose) and dried fruits like figs or dates, generating a beverage with a blond to yellowish color, acidic and slightly alcoholic [14]. The origin of water-kefir is not fully clear, with some descriptions of similar grains reported at the end of the XIX century in Europe coming from the Crimea but there are also reports of sources in Mexico [15]. The fermentations are typically performed at household level and grains are traditionally passed hand in hand [16].

Similar to milk kefir, the fermentation of water kefir is carried out by a distinct starter cultures attached to predominantly polysaccharide matrix called the kefir grain or tibicos. However, differently to milk kefir, the polysaccharide matrix is mostly composed of dextran produced by a variety of lactic acid bacteria species [17,18]. These are comprised mostly of a consortium of lactic acid bacteria, acetic acid bacteria and yeast [14,16,18,19]. Though, clear distinctions to milk kefir are observed in the composition and microbial populations due to the different carbon and energy source for the fermentation [15,18,20,21], which results in variations of the final fermented product. The fermentation of water kefir results in cell growth and associated polysaccharide formation, organic acids, alcohols, carbon dioxide and aroma compounds that are characteristic to the product [17].

Multiple potential health-benefits have been associated with milk kefir, mainly derived from in vitro and animal studies. These effects include immunomodulatory, cholesterol-lowering, anti-allergic, anti-inflammatory, antitumoral, antimicrobial activity (reviewed in [22]) and effect on the gut microbiota to modulate brain physiology [23], linked to the microbial composition of the fermented product, but also to the presence of bioactive molecules and kefiran [21,23]. Most of the current literature evaluating health effects associated to kefir consumption refers to dairy products, but non-dairy kefir derivatives were also linked to specific bioactivity including anti-inflammatory, antiulcerogenic, antihyperglycemic and antihyperlipidemic potential in rodent models (reviewed in [24]). Considering a relative resemblance in consortia composition and microbial dynamics during the fermentation of milk and water kefir, certain similarities could potentially be expected, however due to the main different in substrate, their potential benefit effect should be verified.

Potential health benefits for dairy and non-dairy kefir are mainly related to specific bioactivity of product components or activities of isolates (reviewed in [25]). For example, antimicrobial activity of kefir derived bioactives originated from sugar-broth fermentation have been demonstrated against *Candida albicans, Salmonella typhi, Shigella sonnei, Staphylococcus aureus* and *Escherichia coli* [26]. Rocha-Gomes et al., 2018 showed that supplemented rats with water kefir had an improved plasma and hepatic lipid profile in comparison to the control [27], whereas streptozotocin-induced diabetic rats treated with water kefir showed an improvement in body weight, glucose, and lipid profiles compared with diabetic rats [28]. Water kefir was also proved to be effective in promoting hepatoprotection in a rat model of acetaminophen-induced toxicity, decreasing levels of liver enzymes [29]. Specific lactobacilli strains isolated from Malaysian Kefir and propagated in brown sugar solutions showed high survival to low (3–4) pH, antioxidant properties and high adherence to intestinal cells [30]. However, most of the current literature assessing beneficial properties of kefir is primarily based on in vitro or animal tests, or makes use of dairy kefir products, so further clinical trials are needed to comprehensively assess the effect of water kefir consumption on specific health parameters in healthy and/or diseases population groups. Some commercial kefirs have more complex, less defined culture mixtures, and the mixes differ depending on the source and geographical origin [18]. Pasteurization is a commonly used process on industrial beverages to maintain its safety and organoleptic properties during shelf life. The roles of the microbiota and of pasteurization in mediating the benefits of water kefir have not been clarified. Therefore, further characterization of commercial kefir products on human microbiota and host-microbiome interplay and the effect of pasteurization are still required.

This study assessed in vitro the effect of two water kefir fermented products, a non-pasteurized and a pasteurized form, on microbial metabolic activity and community composition in an in-vitro gastrointestinal system, using short-term colonic incubations representative of three healthy donors. The protective effect of microbial fermentation products derived from the interaction between kefir and gut microbiota on inflammation-induced intestinal epithelial barrier disruption and cytokine production was further evaluated in a co-culture of intestinal epithelial and macrophage-like cells. Incorporating human interindividual variability, microbiota-microbiota and host-microbiota interplay along with pasteurized products representing paraprobiotics (non-viable probiotics) and postbiotics (metabolites and/or cell wall components released by probiotics) in tightly controlled in vitro conditions, provides a better understanding of the potential effects of water kefir on human gut microbiota and intestinal function.

## 2. Materials and Methods

### 2.1. Test Products

All chemicals were obtained from Merck (Darmstadt, Germany) unless stated otherwise. The products tested in this research were obtained from Nestlé and consisted of fermented beverages (water kefir) made with two starter cultures.

Water kefir products were prepared in lab scale simulating home-based products. The grains were obtained from two different sources. Grains referred as K1 were obtained from a household source in Lausanne, Switzerland. The second starter culture were commercial dried water kefir grains obtained from Culture for Health, LLC, Akron, OH, USA. The microbial composition in terms of culturable aerobic mesophilic bacteria, acid lactic bacteria and yeasts of the two kefir cultures is described in Supplementary Table S1. Initially, grains were activated by sub-culturing them twice, in a sugar water supplemented with fig extract: 10.7% (*w*/*w*) of grains and 8.8% (*w*/*w*) of backslope was added to 78.8% (*w*/*w*) of cane sugar solution and 0.5% (*w*/*v*) fig extract, then left at room temperature for 2 to 3 days. Sucrose solution was generated consisting 5% (*w*/*w*) of cane sugar (Migros, Zürich, Switzerland) in two different mineral waters, (V) or (H) (Nestlé, Vevey, Switzerland) filtered through a 0.2 µm sterile filter. Fig (fruit) extract consisted in a 25% (*w*/*v*) of dried figs (Migros, Switzerland), homogenized mineral water, using an Ultra-Turrax (IKA, Staufen, Germany) then centrifuged 7200× *g* for 20 min (SORVALL RC6+ Thermo Scientific, Waltham, MA, USA). The supernatant was collected and autoclaved (121 °C for 15 min; GETINGE GEV6613) before use. For the kefir production, 10.7% (*w*/*w*) of grains were added to a freshly prepared sucrose solution together with 8.8% (*w*/*w*) of backslope, i.e., liquid media resulting from the sub-culture of the kefir grains. Fermentation was carried out in two phases. First, water kefirs were incubated for 24 h at 30 °C in a glass jar covered with a coffee filter. Second, grains were sieved out using a sieve with 1 mm pores and 0.66% (*w*/*w*) of blueberry juice concentrate (Döhler, Germany) plus 0.74% of raspberry juice concentrate (Döhler, Germany) were added to the resulting water kefir liquor. Bottles were closed and left for a second fermentation at 30 °C for 6 additional hours. At the end of the fermentation samples were pasteurized in a steam oven, at 72 °C for 3 min, and directly put on ice.

The composition of the different products was analyzed by HPLC coupled with a refractive index and UV detector (HPLC RI/UV, Agilent Technologies AG, Basel, Switzerland) using the Aminex 87H column (BioRad, Hercules, CA, USA), including total sugars, sucrose, fructose, glucose, ethanol and glucuronic, citric, lactic and acetic acids. 20 µL of injected volume were separated isocratically with 5 mM H_2_SO_4_ for 45 min with a flow rate of 0.6 mL/min and column temperature of 35 °C. Peaks were integrated and quantified using external standards for each specific compound (Merk-Sigma Aldrich, Burlington, MA, USA) via ChemStation (Agilent Technologies). Glucuronic acid, glycerol, propionic acid and butyric acid were also measured but were below the limit of quantification. Aaerobic mesophilic bacteria (AMB), lactic acid bacteria (LAB) and yeast were quantified as described in Supplementary Table S2.

### 2.2. Fecal Sample Collection and Donor Description

Human healthy donors (*n* = 3) were selected based on the following inclusion criteria: healthy, aged between 18–45 years, no antibiotic or any other drug intake during the last 6 months, no constipation, no pre-or probiotic intake. Samples were obtained in special containers containing an “Oxoid™ AnaeroGen™” bag to limit the sample’s exposure to oxygen. Samples were immediately transferred to the lab for further use.

### 2.3. Experimental Design of Short-Term Incubations

**Figure 1:**
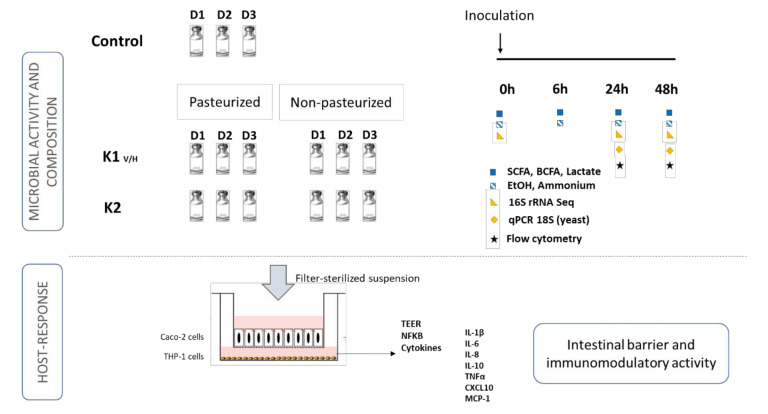
Schematic representation of the experimental set up. SFCA = short-chain fatty acid; BCFA = branched-chain fatty acid; EtOH = ethanol; TEER = transepithelial electrical resistance. D = donor; K = kefir.

A short-term batch assay was performed to assess the effect of a single dose of fermented beverages on the gut microbiota composition and activity of three healthy adults (Figure 1).

Short-term incubations were performed as previously described [31]. Briefly, 57.4 mL of colonic background medium (K_2_HPO_4_ 5.7 g/L; KH_2_PO_4_ 17.9 g/L; NaHCO_3_; 2.2 g/L; yeast extract 2.2 g/L; peptone 2.2 g/L; mucin 1.1 g/L; cysteine 0.5 g/L; Tween80 2.2 mL/L) was mixed with 5.6 mL of different products, resulting in a dose of nutritional components present in products as described in Supplementary Table S2. A control condition containing colonic medium was run in parallel and all the conditions were tested in single reactors, considering the three donors as biological replicates. Then, the reactors were sealed and anaerobiosis was obtained by continuous flushing of the medium with N2 for 10 min. Subsequently, freshly collected fecal sample from three healthy adults were homogenized in anaerobic phosphate buffer (K_2_HPO_4_ 8.8 g/L; KH_2_PO_4_ 6.8 g/L; sodium thioglycolate 0.1 g/L; sodium dithionite 0.015 g/L) in a proportion of 7.5% (*w*/*v*) and 7 mL of the fecal suspension was inoculated in the reactors containing colonic medium with different products.

Samples were collected at 0 h, 6 h, 24 h and 48 h for microbial metabolic activity analysis [pH, gas production, ammonia, short-chain fatty acids (SCFAs) including acetate, butyrate and propionate, branched-chain fatty acid (BCFA), lactate and ethanol] and at 0 h, 24 h and 48 h for community structure analysis (bacterial 16S RNA Illumina sequencing and 18S RNA targeted for total yeasts).

### 2.4. DNA Extraction and Microbial Community Analysis

The DNA was extracted from a pellet of bacterial cells originated from a 1 mL sample after centrifugation for 5 min at 7700× *g*. A Fastprep-24 device (MP BioMedicals, Illkirch, France) was used for homogenization (two cycles of 40 s at 4 m/s). Quality control PCR was conducted using Taq DNA Polymerase with the Fermentas PCR Kit according to the manufacturers’ instructions (Thermo Fisher Scientific, Waltham, MA, USA). The DNA quality was verified by electrophoresis on a 2% (*w*/*v*) agarose gel for 30 min at 100 V.

Microbial community composition was assessed at the start of the experiment and at 24 and 48 h of incubation. Samples were sent out to LGC Genomics GmbH (Berlin, Germany) for next-generation 16S rRNA gene amplicon sequencing of the V3–V4 region. Library preparation and sequencing were performed using an Illumina MiSeq platform with v3 chemistry. The 341F (5′-CCTACGGGNGGCWGCAG-3′) and 785R (5′-GACTACHVGGGTATCTAAKCC-3′) primers were used as previously described [32] with the reverse primer being adapted to increase coverage.

Subsequently, the qPCR for total yeast quantification were performed using a QuantStudio 5 Real-Time PCR system (Applied Biosystems, Foster City, CA, USA), using the primers and conditions described in Supplementary Tables S3 and S4, adapted from [33]. As standard, samples from a pure culture of *Saccharomyces cerevisiae* strain ySR128 was included. The standard curves for all runs had efficiencies between 90 and 105%. Samples were analyzed in technical triplicates and melt curve peaks were checked in each run. Results are reported as log units (18S rRNA gene copies/mL).

### 2.5. Quantification of Total Bacterial Cells by Flow Cytometry

Bacterial cell densities were measured using a BD FacsVerse flow cytometer and live cells were distinguished from damaged cells using SYBR Green (SG; Thermo Fisher, Merelbeke, Belgium)/propidium iodide (PI;Merck KGaA, Darmstadt, Germany) staining, as previously described [34]. Proportional values obtained with Illumina sequencing were converted into absolute quantities by correcting with live bacterial cell counts. Bacterial cells were separated from medium debris and signal noise by applying a threshold level of 200 on the SYTO channel.

### 2.6. Metabolic Analysis

Metabolic parameters were quantified at t0, t24 and t48. In addition, lactate was measured at 6 h (t6). pH measurements were performed using a Senseline pH meter F410 (ProSense, Oosterhout, The Netherlands). Gas formation was measured using a pressure meter to which a needle was connected (Hand-held pressure indicator CPH6200; Wika, Echt, The Netherlands). Gas phase composition was analyzed using a compact GC (Global Analyser Solutions, Breda, The Netherlands), equipped with a Molsieve 5A pre-column and Porabond column (for CH_4_, O_2_, H_2_, N_2_), a Rt-Q-bond pre-column and column (for CO_2_, N_2_O and H_2_S), and a thermal conductivity detector. SCFA (acetate, propionate, and butyrate) and BCFA (isobutyrate, isovalerate, and isocaproate) were determined as described previously [35]. Lactate production was assessed with a kit (R-Biopharm, Darmstadt, Germany) according to manufacturer’s instructions.

### 2.7. Cell Culture

Routine maintenance of cells was performed as described [36]. Briefly, Caco-2 cells (HTB-37; American Type Culture Collection, Manassas, VA, USA) were maintained in Dulbecco’s Modified Eagle Medium (DMEM) containing glucose and glutamine and supplemented with HEPES and 20% (*v*/*v*) heat-inactivated (HI) fetal bovine serum (FBS). THP1-Blue™ were obtained from InvivoGen (Belgium) and consisted in stably transfected cell line with a reporter construct expressing a secreted alkaline phosphatase (SEAP) gene under the control of a promoter inducible by the transcription factor nuclear factor kappa B (NF-κB). THP1-Blue were maintained in Roswell Park Memorial Institute (RPMI)1640 medium containing glucose and glutamine, supplemented with HEPES, sodium pyruvate and 10% (*v*/*v*) HI-FBS. Cells were incubated at 37 °C in a humidified atmosphere of air/CO_2_ (95:5, *v*/*v*).

The Caco-2/THP-1 co-culture was done as previously described [36]. Caco-2 monolayers were differentiated for 14 days on 24-well semi-permeable supports (7.5 × 10^4^ cells/cm^2^), until a functional cell monolayer with a transepithelial electrical resistance (TEER) above 500 ohm Ω cm^2^ was obtained. 48 h before the start of the co-culture, THP1-Blue™ cells were seeded in 24-well plates and stimulated for 48 h with phorbol 12-myristate 13-acetate (PMA) at a concentration of 100 ng/mL to induce macrophage-like phenotype in THP1 cells [37]. After PMA removal, Caco-2-bearing supports were placed on top of PMA-THP1-Blue™ cells, followed by apical treatment with either complete medium or filter-sterilized (0.22 µm) colonic suspensions collected after 24 h and 48 h of incubation. All treatments were done in biological triplicate. TEER was assessed at timepoint 0 h and after 24 h of co-culturing. After 24 h of incubation, the basolateral medium was replaced by RMPI supplemented with ultrapure LPS (Escherichia coli K12, InvivoGen, 10 ng/mL) to disrupt the barrier or left untreated. After 6 h, basolateral medium was collected for cytokine quantification and NF-κB activity measurements. Cytokines were measured using the Luminex technology; while secreted embryonic alkaline phosphatase (SEAP) activity was determined by using the QUANTI-Blue reagent (InvivoGen), following manufacturer instructions.

### 2.8. Data Analysis

Bioinformatics analysis of amplicon data was performed as previously described [38]. Briefly, the mothur software package (v.1.33.3) and guidelines were used to process the amplicon data generated by LGC Genomics. In short, after assembling forward and reverse reads, contigs with a length between 441 and 467 bases were aligned to the mothur formatted silva_seed release 119 alignment database, trimmed between positions 6388 and 25316, to be compatible with the 341F/785R primers. After removing non-aligning sequences as well as sequences containing homopolymer stretches of more than 12 bases, sequences were pre-clustered allowing up to 4 nucleotide differences. UCHIME was applied to remove chimera. Subsequently, sequences were classified, by means of a naive Bayesian classifier, against the RDP 16S rRNA gene training set, version 14 with an 80% cut-off for the pseudobootstrap confidence score. All sequences that were classified as Eukaryota, Archaea, Chloroplasts and Mitochondria were removed and only bacterial sequences were retained. Also, if sequences could not be classified at all [even at (super)Kingdom level] they were removed. Sequences were binned into Operational Taxonomic Units (OTU’s) within each order identified by the preceding classification step. An OTU is defined in this manuscript as a collection of sequences with a length between 402 and 427 nucleotides that are found to be more than 97% similar to one another in the V3-V4 region of their 16S rRNA gene after applying Opticlust clustering [39,40,41,42]. Taxonomy was assigned using the RDP version 16 and silva.nr_v123 database [39,43,44]. The shared file, containing the number of reads observed for each OTU in each sample, was loaded into Microsoft^®®^ Excel^®®^ 2016 MSO (16.0.11901.20070) (Microsoft Corporation, Redmond, WA, USA). Reads occurring only 5 times in all samples were removed, as they were supposedly artefacts or bacteria that were not having any biological impact. For the most abundant OTUs, the sequences retrieved from 3% dissimilarity level fasta file obtained in mothur were classified through the RDP web interface using the RDP SeqMatch tool. The database search was restricted to type strains with only near-full-length and good quality sequences. The sequences were blasted in NCBI against the 16S rRNA gene sequences, selecting only type material, with optimization of the BLAST algorithm for highly similar sequences (accession date: December 2018) [39,43,45]. Although identification to the species level based on short 300 bp reads may involve some ambiguity, the most likely species classification of a few interesting OTUs is reported in the results sections. If two or more species were identified with percentages above 95%, both are presented in the text. In the event of inconsistencies in the results of the RDP SeqMatch tool and NCBI BLAST, no species level classification is provided.

To get a general overview of treatments on microbiota, 16S rRNA raw gene sequences were analyzed using Calypso software Version 8.84. Data were normalized by total sum (TSS) and transformed by square root (Hellinger Transformation). Taxa with less than 0.01% abundance or less than 100 reads were removed.

To take interindividual differences into account, microbial levels at family and OTU for each treatment were normalized by subtracting the value of the control condition for every donor separately. Besides donor effect, the effects of the different treatment parameters were calculated following a similar calculation. In brief, values from pasteurized samples were subtracted from values obtained from non-pasteurized samples per donor to evaluate the pasteurization effect and values from K1 sample were subtracted from the corresponding K2 sample per donor to assess type of kefir effect. The obtained values for the different conditions were averaged over the three donor and *t*-tests were performed to determine statistical significance between different conditions versus the control.

For each microbial metabolic marker (pH, gas production, SCFA, BCFA, lactate) and qPCR data, changes in production or counts between 0 and 6 h (Δ6 h), 6 and 24 h (Δ24 h), and 24 and 48 h (Δ48 h) were calculated. As each variable showed a different trend on maximum changes depending on time, selected values of maximum differences were used for visual representation. After testing for normality (Shapiro Wilk test), one-way ANOVA test was used to determine significance, with Tukey’s multiple comparison test as post-hoc. Arithmetic mean and standard error of the mean (SEM) were used as descriptive statistics. Data were also analyzed after normality test, using two-way ANOVA, including repeated measures on different time points, and checking the interaction between treatment and time. In this case, Dunnett’s multiple comparison tests was used to determine the significance between different treatments and the control condition. To compare the overall changes of kefir treatments versus the control, paired *t*-tests were used. A General Linear Model was used to determine the effect of donor, pasteurization, kefir culture and water used for kefir preparation as factors.

Clustvis software was used to perform the Principal Component Analysis (PCA) plots on microbial metabolites. Briefly, unit variance scaling is applied to rows and SVD with imputation was used to calculate principal components, represented in the X (PC1) and Y (PC2) axis. Prediction ellipses were drawn at 95% of confidence interval.

For cell culture assays, treatment samples were compared to the control samples using two-way ANOVA with Sidak’s multiple comparisons test. Significant differences are represented by (*), (**), (***) and (****) representing *p* < 0.05, *p* < 0.01, *p* < 0.001 and *p* < 0.0001, respectively.

Statistical analysis was performed in GraphPad Prism version 8.2.0 (435) for Windows (GraphPad Software, San Diego, CA, USA) or Minitab version 18.1 (Minitab GmbH, Munich, Germany). All formal hypotheses were conducted on the 5% significance level (α = 0.05).

### 2.9. Ethics

Fecal samples of the healthy donors were collected according to the ethical approval of the University Hospital Ghent (reference number B670201836585).

## 3. Results

### 3.1. Water Kefir Beverages Induced a Change in Metabolic Microbial Activity

In general, water kefir, pasteurization and donor had a significant effect on most of the microbial metabolic markers, while type of water used to prepare the kefir only affected lactate, ammonia and BCFA (Table 1).

**Figure 2:**
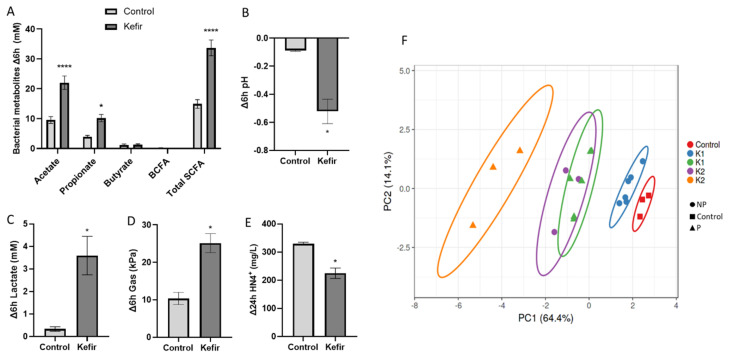
Effect of kefir supplementation on bacterial-derived metabolites. (**A**–**E**) Bars represent the delta change at 6 h (Δ6 h) for SCFA and BCFA (**A**,**B**), pH (**C**), lactate (**D**) and gas (**E**), and at 24 h (Δ24 h) for ammonia. Values are expressed as mean ± SEM of control (*n* = 3) or kefir-treated (*n* = 9) reactors. Significant differences are marked with asterisks (* *p* < 0.05, **** *p* < 0.0001). (**F**) Principal Component Analysis plot of microbial metabolic markers, including absolute values of pH, gas, lactate, SCFA, BCFA and ammonia at 6, 24 and 48 h. Ellipses are drawn at 95% confidence interval.

Considering all the kefir products independently of the kefir source or pasteurization, total short-chain fatty acids were consistently increased by kefir treatments (Δ_6 h_ 33.7 ± 3 mM) compared to the control (Δ_6 h_ 14.9 ± 0.8 mM) (Figure 2A). This effect was observed in short-term incubation (Δ_6 h_) but was not significant at longer incubation times (Δ_24 h_). Changes in total SCFA were primarily caused by the major metabolite acetate, which showed the same trend described above (Figure 2A, Table 1). Propionate was also significantly increased by kefir (Δ_6 h_ 10.2 ± 1.4 mM) compared to the control condition (Δ_6 h_ 3.9 ± 0.3 mM) (Figure 2B). Butyrate levels were only increased by kefir at 24 h (Δ_24 h_ 4.2 ± 0.5 mM) compared to control (Δ_24 h_ 2.7 ± 0.5 mM) (*p* < 0.04). BCFA did not differ at Δ6 h or Δ24 h. Consistently with observed changes in SCFA, a decrease in pH (Δ_6 h_ −0.5 ± 0.09) and increased levels of lactate (Δ_6 h_ 3 ± 0.6 mM) and gas production (Δ_6 h_ 25.1 ± 2.9 kPa) were observed in kefir-exposed reactors (Figure 2C,E). As a marker of proteolytic fermentation, ammonia levels were significantly reduced by kefir (Δ_24 h_ 289.4.7 ± 18.5 mg/L), compared to the control condition (Δ_24 h_ 380.4 ± 5.3 mg/L) (Figure 2F).

When analyzing the pasteurization effect on microbial metabolic products, acetate and propionate were higher in pasteurized samples than in non-pasteurized ones at 6 h (*p* < 0.001, Table 1). Butyrate levels were significantly increased in pasteurized samples at 24 h (Table 1), with no significant effects at other time points. Contrarily, ammonia was significantly lower in reactors exposed to pasteurized samples at 24 h (Table 1).

When considering each product independently, a different response was observed for K1 and K2 (Table 1), with the highest effect on microbial metabolic activity observed for K2_P (Figure 2F, Supplementary Figure S1). Consistently, K2_P significantly increased acetate (Δ_6 h_ 48.3 ± 1.7 mM), propionate (Δ_6 h_ 13.8 ± 2 mM) and butyrate (Δ_24 h_ 6.8 ± 0.5 mM) and reduced proteolytic fermentation markers BCFA (Δ_24 h_ 0.4 ± 0.1 mM) and ammonia (Δ_24 h_ 144.1 ± 4.5 mg/L), compared to control condition (Supplementary Figure S1).

Type of water used in kefir preparation influenced BCFA production, with higher levels observed in H (1.3 ± 0.4 mM) compared to V (0.5 ± 0.1 mM) (Table 1) and lactate (Δ_6 h_ H = 0.05 ± 0.6 mM; V = 3.5 ± 0.9 mM) (Table 1).

### 3.2. Water Kefir Beverages Induced a Shift in Microbial Structure of Healthy Adults

**Figure 3:**
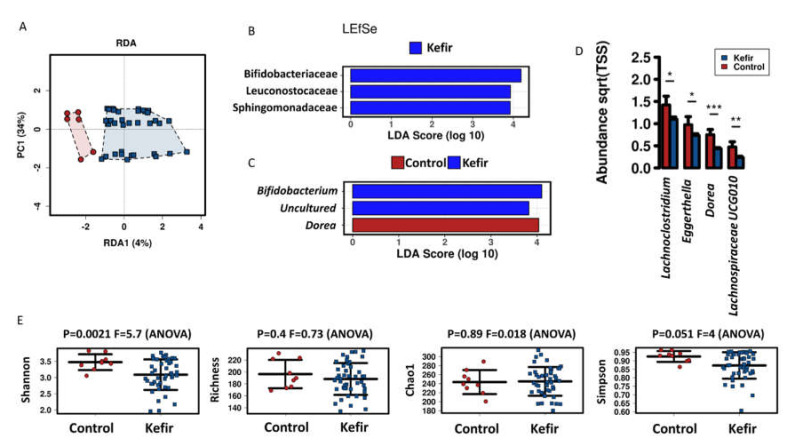
Effect of kefir on microbial communities in vitro. (**A**) Redundancy analysis (RDA) plot of microbial community of control and kefir-exposed reactors, including three different donors and two time points (24 h and 48 h). Linear discriminant effect size (LEfSe) analysis at family (**B**) and genus (**C**) level. (**D**) Bar plot of the abundance at genus level of statistically significant [*p* < 0.05 (*), *p* < 0.01 (**), *p* < 0.001 (***)] differences between control and kefir condition. (**E**) Strip charts representing Shannon, Richness, Chao1 and Simpson’s index at OTU level. Sqrt(TSS) = square root total sum of squares.

In our setup, the main effects on microbial communities in vitro were time and donor (Supplementary Figure S2). Overall, the microbial communities from control condition and kefir treatments clustered separately (Figure 3A), however, the effect of kefir starter culture was not significant using a multivariate analysis (Adonis based on Bray-Curtis distance *p* = 0.457).

When performing a linear discriminant analysis effect size (LEfSe) analysis at family level, kefir-treated reactors were enriched (LDA > 3) in *Bifidobacteriaceae*, *Leuconostocaceae* and *Sphingomonadaceae* (Figure 3B) compared to control samples. LEfSe analysis at the genus level showed enrichment of *Dorea* in the control group, while *Bifidobacterium* and uncultured bacterium were enriched in kefir reactors. The abundance of *Lachnoclostridium*, *Eggerthella*, and *Lachnospiraceae_*UCG010 was also reduced by kefir (Figure 3D). At OTU level, Shannon index showed a significant decrease with kefir, whereas other diversity indices were not significantly affected by kefir treatments (Figure 3E).

**Figure 4:**
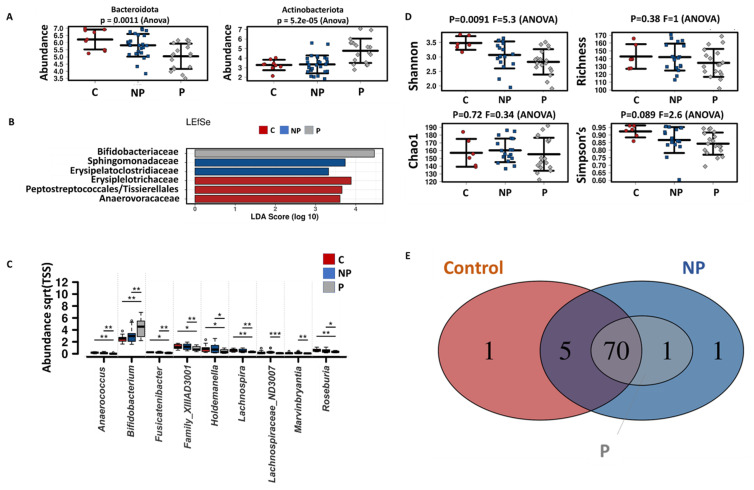
Effet of pasteurization of kefir on microbial modulation in vitro, including three different donors and two time points (24 h and 48 h). (**A**) Strip charts at phylum level of statistically significant differences between non-pasteurized and pasteurized samples. (**B**) Linear discriminant effect size (LEfSe) analysis family level. (**C**) Box plot of the abundance at genus level of statistically significant [*p* < 0.05 (*), *p* < 0.01 (**), *p* < 0.001 (***)] differences between non-pasteurized and pasteurized samples. (**D**) Strip charts of Shannon, Richness, Chao1 and Simpson’s index. (**E**) Core microbiota plot at genus level, showing unique and common features to the different treatment groups. Sqrt(TSS) = square root total sum of squares.

When looking specifically at the effect of pasteurization on microbial communities, specific taxa were differentially modulated. Pasteurized kefir caused a significant enrichment on Actinobacteria phylum and decreased Bacteroidota compared to non-pasteurized products (Figure 4A). At the family level, pasteurization induced an enrichment of *Bifidobacteriaceae* compared to non-pasteurized samples, which contrarily showed a higher abundance of *Sphingomonadaceae* and *Esysipelatoclostridiaceae* (Figure 4B). A reduction of minor genera like *Anaerococcus, Family_XIII_AD3011_group, Fusicatenibacter, Holdemanella, Lachnospira, Lachnospiraceae_ND3007_group, Marvinbryantia* and *Roseburia* was observed in pasteurized samples as compared to non-pasteurized ones. Consistently, *Bifidobacterium* genus was enriched in pasteurized samples (Figure 4C). Shannon index was significantly reduced by kefir treatments, especially from pasteurized samples, while richness, Chao1 and Simpson index were not significantly affected by the treatments (Figure 4D). *Erysipelotrichaceae*_unclassified was a unique feature detected in non-pasteurized samples and *Peptostreptococcus* genus was present only in water kefir-treated reactors (Figure 4E).

When looking at the two specific products used in this research, the main differences observed for kefir were induced by K2, which significantly reduced Desulfobacterota and Bacteroidota phylum compared to K1 and control conditions (Supplementary Figure S3). *Bifidobacteriaceae* enrichment was associated with K2 treatment, mainly due to changes in OTU2 (*Bifidobacterium adolescentis*/*faecalis*) (Supplementary Tables S5 and S6).

### 3.3. Pasteurization Had a Significant Effect on Ethanol Production and Yeast Levels

**Figure 5:**
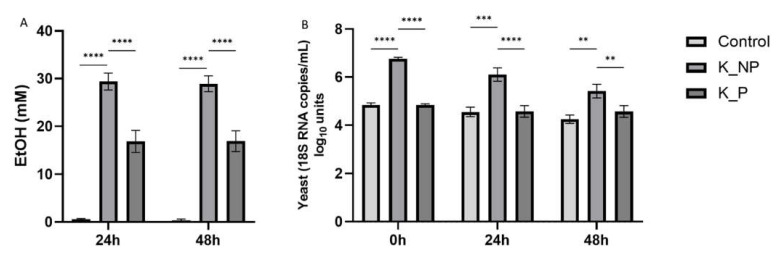
Effect of kefir products on ethanol and yeast levels. (**A**) Bars represent mean ± SEM (*n* = 3) of EtOH (mM) levels different reactors exposed to non-pasteurized and pasteurized kefir products for 24 and 48 h. (**B**) Bars represent the mean ± SEM of yeast 18S RNA copies/mL quantified by qPCR at different time points. Significant differences between control condition and treatments or between non-pasteurized and pasteurized products are marked by (**), (***) and (****) *p* < 0.05, *p* < 0.01, *p* < 0.001 and *p* < 0.0001, respectively.

In the control condition, EtOH levels were lower (0.6 ± 0.2 mM, 24 h) than in water kefir-treated reactors (23.1 ± 8.8 mM, 24 h) (*p* < 0.0001). The highest levels of EtOH were quantified on colonic incubations exposed to non-pasteurized products (28.9 ± 5 mM, 24 h), with the highest values observed for K1. Pasteurized samples showed a consistent decrease in EtOH levels (16.9 ± 6.9 mM; 24 h) compared to non-pasteurized ones (Figure 5A) (*p* < 0.001).

Quantification of 18S RNA copies showed similar values for control condition at the different time points (4.6 ± 0.2 copies/mL log_10_ units), while non-pasteurized products significantly increased 18S RNA copies (K1 = 5.8 ± 0.5 copies/mL log_10_ units; K2 = 6.7 ± 0.1 copies/mL). Pasteurization significantly reduced 18S RNA copies, especially in kefir K1, which shows similar levels to control (4.4 ± 0.2 copies/mL log_10_ units), while pasteurized K2 still had higher levels at 24 h and 48 h compared to the control condition (5.5 ± 0.06 copies/mL log_10_ units). The amount of yeast in non-pasteurized product decreased over time.

### 3.4. Water Kefir Diminished Inflammation-Induced Intestinal Epithelial Barrier Disruption

**Figure 6:**
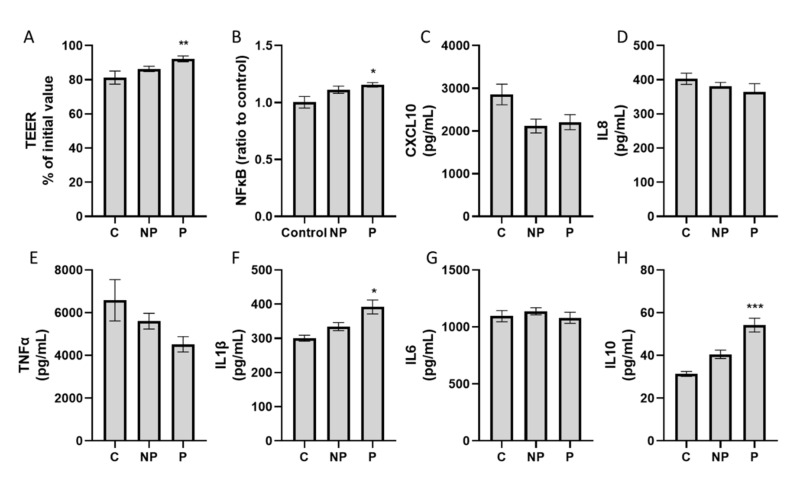
Effect of kefir on the intestinal barrier and immunomodulation. Bars represent the mean ± SEM (n ≥ 3) for TEER percentages of the initial value (**A**), NF-κβ activity of THP1 cells (**B**), CXCL10 (**C**), IL8 (**D**), TNFα (**E**), IL1β (**F**), IL6 (**G**) and IL10 (**H**). Significant differences between control condition and treatments marked by (*), (**) and (***) for *p* < 0.05, *p* < 0.01 and *p* < 0.001, respectively.

Cells exposed to microbial metabolites from kefir-treated reactors had significantly higher (*p* = 0.019) percentages of TEER (89 ± 2%) compared to the initial values than the control condition (81 ± 6%). Pasteurization increased the effect of kefir products on TEER enhancement (*p* = 0.014), with values of 92 ± 3% and 86 ± 3% for pasteurized and non-pasteurized products, respectively, with pasteurized K2 showing the highest increase on TEER values (93 ± 4%) (Figure 6A, Supplementary Figure S4).

### 3.5. Immunomodulatory Activity of Water Kefir at the Intestinal Level

In general, when considering all treatments together, NF-κB activity was not significantly modified by kefir (Figure 6). However, colonic batch suspensions of all pasteurized products increased NF-κB activity, with the strongest effects seen for K1 (0.7 ± 0.03 OD_630_) (Supplementary Figure S4).

Overall, pasteurization had a significant effect on modulatory activity of kefir products. Cells exposed to pasteurized kefir products significantly increased IL10 (kefir = 53 ± 0.7 pg/mL; control = 31 ± 0.2 pg/mL, *p* < 0.001) and IL1β (kefir = 389 ± 4.7 pg/mL; control = 300 ± 1.5 pg/mL, *p* = 0.01) (Figure 6).

The effect of different parameters, including inoculum, pasteurization and water on epithelial barrier integrity, NF-κB activation and immunomodulatory activity in each donor is presented in supplementary Figures S5 and S6.

## 4. Discussion

Modulation of composition and metabolic activity of gut microbiota from three healthy donors by different kefir products was evaluated using a short-term simulation of the colonic fermentation process.

Despite significant interindividual differences in basal microbiota, kefir supplementation showed a consistent potential to modulate microbial activity in vitro, likely by adding fermentative substrates and microorganisms to the background colonic media. Overall, all products increased short-chain fatty acids and lactate and reduced ammonia production, with the highest effect observed for pasteurized K2, which increased health-promoting propionate and butyrate. Major SCFAs, such as acetate, propionate and butyrate, are important metabolites for intestinal homeostasis, metabolic control, and immune programming [46,47,48,49]. Specifically, butyrate is the primary source of energy for colonocytes and is involved in maintaining the epithelial barrier function [50,51]. Increased fecal SCFAs were also observed in human kefir-intervention studies [52]. Along with increases in beneficial SCFAs, kefir reduced by-products of protein and nitrogen metabolism. Urease producing bacteria and glutaminase positive bacteria significantly mediate ammonia production in the colon, which is known to have negative effects on epithelial health [53]. Taken together, these results potentially link kefir intake with positive regulation of the colonic microenvironment, inducing beneficial SCFAs production while reducing detrimental proteolytic fermentation compounds.

Linked to microbial metabolic activity, changes in ecosystem structure were also detected, with the *Bifidobacteriaceae* family being consistently increased by water kefir. *Bifidobacterium* spp. are early colonizers of the human gut and are associated with a healthy condition [54]. Reduced *Bifidobacteriaceae* populations have been described in different pathological conditions such as allergy or inflammatory bowel disease [54,55,56]. Water kefir-related increases of *Bifidobacteriaceae* in our setup likely explain the increase of acetate and lactate production, which can cross-feed commensal butyrate or propionate-producing anaerobes [57]. This in turn may explain the finding of increased butyrate at 24 h, whereas acetate is not different from control. Pasteurized products had a more substantial bifidogenic effect than non-pasteurized counterparts, therefore the observed increase in *Bifidobacterium* spp might be linked to the stimulation of resident bifidobacterial populations. This observation also suggests that the thermal degradation of water kefir yeast and bacterial microbial populations makes available different carbon sources that promote *Bifidobacterium* growth, such yeast glycans [58,59]. Quantification of yeasts by qPCR and significant reduction of ethanol levels evidenced a degradation of yeasts during pasteurization process and enzymatic machinery in *Bifidobacterium* spp. have been reported to selectively uptake carbohydrates from glycans. Significant increases in the relative abundance of Actinobacteria have been reported in a parallel-group, randomized, controlled clinical trial setting with patients with metabolic syndrome receiving milk kefir compared to unfermented milk during 12 weeks [60], a trait also observed in this study. Specific components of water kefir responsible for its effects in animal and human intervention studies have not been fully determined but levan increased bifidobacteria levels in an in vivo rat model supplemented for 90 days with different doses of levan [61]. Levan is a water-soluble fructose polymer produced by bacteria and archaea via secreted or cell-wall anchored, extracellular levansucrases [62]. In our research, OTU2, related to *Bifidobacterium adolescentis*/*faecalis* was significantly induced by water kefir, especially by pasteurized samples, extending the paraprobiotic/postbiotic application of water kefir products and suggesting a species-specific bifidogenic effect depending on background host microbiota. In parallel to *Bifidobacterium* spp. stimulation, kefir induced a decrease in the abundance of *Dorea* genus, a commensal member of the gut microbiota, which is over-represented in individuals with asthma, type 2 diabetes [9,10] and obese mice [63].

Multiple mechanisms have been previously described for the modulation of gut microbiota by probiotics or paraprobiotics to beneficially affect host health, including microbe-microbe interaction of kefir strains with intestinal microorganisms, microbe-host interaction with intestinal epithelium, modulation of mucus production or epithelial barrier function, local or distal immunomodulation or effects on gut-organs communication (e.g., gut-brain, gut-liver, gut-lung axis) [64]. We demonstrated the protective effects of water kefir products on inflammation-induced intestinal barrier disruption, with the most potent effect observed in pasteurized product K2. This result is in line with the highest modulatory activity of pasteurized products on gut microbiota but can also be a cause of the increased release of bioactive molecules derived from water kefir yeast and bacteria during inactivation. The increased SCFA production by water kefir, especially pasteurized K2 kefir, is a potential molecular mechanism for the enhanced gut barrier [65,66].

Consistently, pasteurized kefirs enhanced intestinal barrier function and promoted anti-inflammatory IL-10 production. Microbial metabolites, like butyrate, acetate or propionate, but also yeast-derived molecules could be involved in the upregulation of IL-10 observed in our model. IL-10 is a central cytokine in maintaining mucosal tolerance, signaling different lymphocyte T subpopulations, downregulating TNFα, and influencing selective colonization of the gut [67,68] and it has been proposed as a target for reducing gut inflammation [51]. Butyrate has been shown to enhance IL-10 secretion in human monocytes, and different *Saccharomyces* strains also showed a stimulatory effect on IL-10 production by human peripheral blood mononuclear cells [69]. The same authors proposed that yeast regulation of gut inflammation in a mouse model may be mediated by reinforcement of the intestinal barrier, a mechanism supported by a recent study in piglets that showed how yeast culture improved the expression of tight junction proteins [70].

Pasteurized water kefir also induced activation of NF-κB and secretion of IL-1β, both usually considered pro-inflammatory markers. NF-κB regulates inflammation, immune responses, cell proliferation, survival, and apoptosis, and it is considered a key signaling molecule in maintaining epithelial barrier and immune homeostasis [71], while dysregulated IL-1β production has been linked to multiple inflammatory conditions [72]. However, a complex cross-regulation between pro- and anti-inflammatory cytokines occurs physiologically in the gut. For example, intestinal macrophage-derived IL-1β produced in response to commensal stimuli are key in maintaining oral tolerance and generating homeostasis-promoting regulatory T cells [72]. The effect of pasteurization on modulation of different cytokines suggests a major effect of water kefir processing on shifting the immune response in vitro, and due to the complex microbial nature of the product, further characterization of immunomodulatory molecules is proposed.

## 5. Conclusions

Most of the research involving pre and probiotic properties of kefir is based on milk kefir products, while this research provides novel information on potential modulatory effect of water kefir on human gut microbiota. Our study using an artificial colon set up suggests beneficial effects of water kefir consumption through SCFA production, bifidobacteria induction and epithelial barrier enhancement, while limitations on translatability and interindividual variability will require validation in clinical settings. Intriguingly, the observed benefits are enhanced by pasteurization, supporting water kefir derived products as paraprobiotics/postbiotics with potential health benefits and the added value of extended shelf life and stability of the product.

## Figures and Tables

**Table 1:** Summary results of a General Linear Model of different metabolites and variables of the study, including donor, product pasteurization, kefir inoculum and type of water used for kefir preparation.

		Δ6 h Acetate (mM)	Δ6 h Propionate (mM)	Δ24 h Butyrate (mM)	Δ24 h BCFA (mM)	Δ6 h SCFA (mM)	Δ24 h Ammonia (mg/L)	Δ6 h Lactate (mM)	Δ6 h pH	Δ6 h Gas (kPa)
	R-sq	92.93%	89.69%	79.61%	50.06%	98.83%	98.03%	23.33%	91.99%	88.86%
Pasteurization	*p*-Value	<0.001	0.001	<0.001	0.20	<0.001	<0.001	0.91	<0.001	0.15
	NP	19.72 ± 1.77	9.25 ± 1.02	3.8 ± 0.26	1.10 ± 0.27	30.54 ± 2.22	262.92 ± 13.6	1.84 ± 1.29	−0.36 ± 0.05	26.20 ± 2.87
	*p*	28.25 ± 2.59	12.58 ± 1.54	5.3 ± 0.56	0.77 ± 0.21	42.27 ± 2.49	192.45 ± 15.40	1.71 ± 0.31	−0.70 ± 0.08	28.72 ± 3.05
Donor	*p*-Value	<0.001	<0.001	0.009	0.087	<0.001	0.003	0.55	0.04	<0.001
	A	24.19 ± 2.82	8.45 ± 0.94	5.3 ± 0.72	1.04 ± 0.25	33.46 ± 3.69	216.42 ± 25	2.67 ± 1.16	−0.56 ± 0.12	23.87 ± 2.00
	B	19.12 ± 1.72	15.29 ± 1.74	4.6 ± 0.51	1.23 ± 0.42	35.52 ± 3.39	234.41 ± 22.93	1.36 ± 0.56	−0.46 ± 0.09	22.00 ± 2.00
	C	28.65 ± 3.78	8.95 ± 0.45	3.8 ± 0.53	0.53 ± 0.06	40.23 ± 4.17	232.21 ± 23.94	1.30 ± 1.52	−0.57 ± 0.12	36.50 ± 3.19
Kefir	*p*-Value	<0.001	0.001	<0.001	0.37	<0.001	<0.001	0.02	<0.001	<0.001
	K1	18.94 ± 1.50	9.11 ± 1.02	3.5 ± 0.25	1.07 ± 0.23	19.69 ± 1.88	266.67 ± 10.72	3.60 ± 0.86	−0.34 ± 0.04	22.04 ± 2.00
	K2	29.04 ± 4.28	12.72 ± 2.05	5.6 ± 0.71	0.80 ± 0.07	19.90 ± 5.60	188.7 ± 17.81	−0.04 ± 0.66	−0.72 ± 0.11	32.88 ± 4.61
Water	*p*-Value	0.30	0.41	0.44	0.02	0.003	0.021	0.03	0.47	0.91
	H	24.76 ± 2.20	11.30 ± 1.52	4.7 ± 0.42	1.33 ± 0.39	20.48 ± 2.78	233.68 ± 16.07	0.05 ± 0.59	−0.55 ± 0.06	29.00 ± 2.95
	V	23.21 ± 2.52	10.53 ± 1.40	4.4 ± 0.49	0.54 ± 0.08	19.12 ± 2.35	221.68 ± 16.22	3.51 ± 0.89	−0.51 ± 0.08	25.92 ± 2.64

## Data Availability

The data that support the findings of this study are available from the corresponding authors, upon reasonable request.

## Supplementary Materials

The following are available online at https://www.mdpi.com/article/10.3390/nu13113897/s1. Figure S1: Short-term effect of different kefir products on microbial activity of healthy donors. Figure S2: Discriminant Analysis of Principal Components and Adonis based on Bray-Curtis distance for time and donor at family level. Figure S3: Effect of different starting cultures of water kefir on microbial structure in vitro. Figure S4: Effect of different products on epithelial barrier and immune modulation. Figure S5: Effect of colonic batch suspensions on transepithelial electrical resistance (TEER) of the Caco-2/THP1-BlueTM co-cultures (A) and NFκβ activity of THP1-Blue™ cells (B), including interindividual differences. Figure S6: Effect of colonic batch suspensions on secretion of IL-6 (A), IL-10 (B), IL-1β (C), TNF-α (D), CXCL10 (E) and IL-8 (F) including interindividual differences. Table S1: Composition of different products used in this research, including sucrose, fructose, lactic acid, acetic acid, ethanol, glucose and total sugar content, and bacterial quantification of and aerobic mesophilic bacteria (AMB), lactic acid bacteria (LAB) and yeasts. Table S2: Experimental conditions for quantification of aerobic mesophilic bacteria (AMB), lactic acid bacteria (LAB) and yeast. Table S3: Primers used for yeast quantification by qPCR. Table S4: Conditions used to quantify yeast populations by qPCR. Table S5: Average normalized microbial shifts and effects of the different treatment parameters (pasteurization, starting culture and type of water) on the most abundant OTUs detected after 24 h of incubation. Table S6: Average normalized microbial shifts and effects of the different treatment parameters (pasteurization, starting culture and type of water) on the most abundant OTUs detected after 48 h of incubation.
