# Supplementary material for: Water Kefir and Derived Pasteurized Beverages Modulate Gut Microbiota, Intestinal Permeability and Cytokine Production In Vitro
Source: Nutrients. 2021 Oct 29;13(11):3897. doi: 10.3390/nu13113897 (PMC8625349; doi:10.3390/nu13113897)
Supplement: Supplementary file 1 [file nutrients-13-03897-s001.zip › nutrients-1410282-supplementary.pdf]

Supplementary Materials

Supplementary Tables

Table S1. Composition of different products used in this research, including sucrose, fructose, lactic acid, acetic acid, ethanol, glucose and total sugar content, and bacterial quantification of and aerobic mesophilic bacteria (AMB), lactic acid bacteria (LAB) and yeasts.

			**g/L**							**CFU/mL**		
K_{efir}	$P_{asteurization}$	W_{ater}	Sucrose	Fructose	Lactic acid	Acetic acid	Ethanol	Glucose	Total sugar	AMB	LAB	Yeast
K1	No	V	11.57 ± 0.26	4.08 ± 0.04	0.39 ± 0.01	0.93 ± 0.02	14.26 ± 0.02	0.90 ± 0.07	16.55 ± 0.16	$1.7x10^6$	$2.0 x10^6$	$6.85x10^5$
K1	No	H	12.29 ± 0.25	4.62 ± 0.01	0.47 ± 0.01	0.82 ± 0.01	12.40 ± 0.01	1.34 ± 0.08	18.26 ± 0.17	$2.5 x10^6$	$1.2 x10^7$	$6.25x10^5$
K1	Yes	V	13.99 ± 0.03	4.82 ± 0.02	0.40 ± 0.01	0.94 ± 0.02	12.54 ± 0.01	1.11 ± 0.01	19.91 ± 0.04	<10	20	<10
K1	Yes	H	15.23 ± 0.02	5.38 ± 0.01	0.45 ± 0.01	0.81 ± 0.02	11.20 ± 0.01	1.37 ± 0.01	21.99 ± 0.03	30	100	<10
K2	No	V	36.26 ± 0.38	4.01 ± 0.02	0.24 ± 0.01	0.72 ± 0.01	3.90 ± 0.01	2.79 ± 0.05	43.06 ± 0.31	$9.2x10^6$	$4.1x10^5$	$1.04x10^6$
K2	Yes	V	41.14 ± 0.11	3.74 ± 0.05	0.23 ± 0.04	0.73 ± 0.01	2.41 ± 0.03	3.06 ± 0.03	47.94 ± 0.03	550	30	250

Table S2. Experimental conditions for quantification of aerobic mesophilic bacteria (AMB), lactic acid bacteria (LAB) and yeast. Samples were serially diluted in PBS before being plated in selected media, and incubated at 30°C. MRS: De Man, Rogosa and Sharpe agar. YPD: yeast extract, peptone, dextrose media. PCA: plate count agar. AN: anaerobic. AE: aerobic.

	Medium	Selection agent	Oxygen conditions	Incubation time (day)
LAB	MRS	21.6 µg/mL natamycin	AN	2
Yeast	YPD	100 µg/mL chlortetracycline	AE	2
AMB	PCA	None	AE	2

Table S3. Primers used for yeast quantification by qPCR.

Primer name	Sequence 5’ – 3’	Reference
FF390	CGATAACGAACGAGACCT	Prevost-Boure (2011)
FR1	AICCATTCAATCGGTAIT	

Table S4. Conditions used to quantify yeast populations by qPCR.

Program	Cycle	Temperature (°C)	Heating (hh:mm:ss)	Ramp (°C/s)
Pre-incubation	1	95	00:10:00	1.6
Amplification	40	95	00:00:15	1.6
		50	00:00:30	1.55
		72	00:00:30	1.6
Melting curves	1	95	00:00:15	1.6
		60	00:01:00	1.55
		75	00:00:15	0.075

Table S5. Average normalized microbial shifts and effects of the different treatment parameters (pasteurization, starting culture and type of water) on the most abundant OTUs detected after 24h of incubation. Positive values indicate enrichment by the treatment and are indicated in green. Statistical significance of an enrichment is indicated in bold (p<0.05). K1 = starting culture 1; K2 = starting culture 2; NP = non-pasteurized; P = pasteurized.

Phylum	Family	OTU	Closely related species	Average normalized microbial shifts						Treatment effects		
				K1 H		K1 V		K2 V		P-NP	K2-K1	HB-VB
				NP	P	NP	P	NP	P			
Actinobacteria	*Bifidobacteriaceae*	2	*Bifidobacterium adolescentis (100) / faecale (100)*	0.28	**0.74**	**0.13**	**0.70**	**0.77**	**0.92**	**0.40**	**0.43**	0.09
		11	*Bifidobacterium longum subsp. null (100) / longum subsp. longum (100)*	0.16	0.34	**0.06**	**0.25**	**0.36**	**0.27**	0.09	0.16	0.09
		30	*Bifidobacterium longum subsp. Null (98) / longum subsp. Longum (98)*	0.25	0.53	**0.14**	**0.42**	**0.48**	**0.58**	**0.22**	0.25	0.11
		37	*Bifidobacterium pseudocatenulatum (100) / kashiwanohense (100) / catenulatum (100)*	**0.17**	0.74	**0.16**	**0.69**	0.45	**0.95**	**0.53**	0.28	0.03
	Coriobacteriaceae	8	*Collinsella aerofaciens (100)*	0.06	-0.28	-0.03	-0.40	-0.33	0.15	-0.08	0.13	**0.11**
	Eggerthellaceae	43	*Coriobacteriaceae bacterium WAL 18889 (98), Senegalimassilia anaerobia (97)*	0.05	0.14	0.05	0.14	0.15	0.03	0.02	0.00	0.00
Bacteroidetes	*Bacteroidaceae*	4	*Bacteroides vulgatus (100)*	0.05	-0.24	0.04	-0.16	-0.25	0.09	-0.05	-0.02	-0.04
		6	*Bacteroides uniformis (100)*	0.08	-0.06	**0.05**	**-0.11**	**-0.21**	-0.23	**-0.11**	**-0.19**	0.04
		7	*Bacteroides uniformis (100)*	0.15	-0.04	**0.10**	-0.09	-0.22	-0.27	**-0.14**	**-0.25**	0.05
		13	*Bacteroides eggerthii (100)*	-0.14	-0.12	-0.10	-0.19	-0.11	0.01	0.01	0.10	0.02
		15	*Bacteroides stercoris (100)*	0.26	0.11	0.00	0.11	-0.01	-0.11	-0.05	-0.11	0.13
		19	*Bacteroides plebeius (100)*	**0.65**	0.59	0.12	**0.46**	0.49	0.35	0.05	0.13	**0.33**
		23	*Bacteroides faecis (100) / thetaiotaomicron (100)*	0.35	0.10	0.38	0.26	0.02	-0.03	-0.14	-0.32	-0.09
		31	*Bacteroides thetaiotaomicron (100)*	-0.08	-0.31	0.00	**-0.01**	-0.15	**-0.79**	-0.30	-0.46	-0.19
		33	*Bacteroides ovatus (100)*	0.17	0.01	**0.14**	**-0.18**	**-0.17**	-0.28	**-0.20**	**-0.20**	0.11
		34	*Bacteroides fragilis (100)*	0.09	0.17	0.13	0.21	0.13	0.00	0.01	-0.10	-0.04
		40	*Bacteroides massiliensis (100)*	-0.16	-0.34	-0.23	-0.48	-0.58	-0.58	-0.14	-0.22	0.11
		42	*Bacteroides sp. (82)*	0.87	**0.34**	0.51	0.23	0.41	0.42	-0.27	0.04	0.24
		48	*Bacteroides sp. (77)*	0.14	0.10	0.14	0.05	-0.15	-0.22	-0.06	-0.27	0.03
		49	*Bacteroides coprophilus (97)*	0.37	0.22	0.09	0.05	0.07	0.14	-0.04	0.03	**0.23**
	Porphyromonadaceae	38	*Unclassified Porphyromonadaceae (62)*	0.20	0.24	0.14	0.19	0.09	-0.08	-0.03	-0.16	0.05
	Prevotellaceae	14	*Prevotella sp. (68)*	0.35	0.51	0.09	0.28	0.15	-0.01	0.06	-0.12	**0.24**
		41	*Prevotella sp. (96)*	0.37	0.59	0.47	0.65	0.69	0.59	0.10	0.08	**-0.08**
	Rikenellaceae	17	*Alistipes sp. (85)*	0.23	0.23	0.23	0.21	0.22	0.03	-0.07	-0.09	0.01
		24	*Alistipes finegoldii (100) / onderdonkii (100)*	0.23	-0.06	0.15	0.06	0.10	-0.18	**-0.22**	-0.14	-0.02
		26	*Alistipes putredinis (100)*	-0.05	-0.23	-0.05	-0.23	-0.19	-0.37	**-0.18**	-0.14	0.00
	Tannerellaceae	20	*Parabacteroides distasonis (99)*	-0.20	**-0.23**	-0.12	**-0.24**	-0.13	**-0.43**	**-0.15**	-0.10	-0.04
		28	*Bacteroides merdae (100) , Parabacteroides sp. S448 (100)*	**0.10**	0.07	0.07	-0.13	0.01	-0.11	-0.12	-0.02	0.12
Desulfobacterota	*Desulfovibrionaceae*	62	*Desulfovibrio sp. (96)*	**0.18**	0.14	0.19	0.02	0.10	0.60	0.10	0.25	0.06
Firmicutes	*Acidaminococcaceae*	10	*Clostridium carnis (100) , Phascolarctobacterium faecium (100)*	0.14	-0.08	0.04	-0.24	-0.17	-0.32	**-0.22**	-0.15	**0.13**
	Christensenellaceae	32	*Unclassified Clostridiales (81)*	0.11	0.08	0.14	0.13	0.10	-0.09	-0.08	-0.13	-0.04
	Erysipelotrichaceae	25	*Holdemanella sp. (91)*	0.02	0.03	-0.12	-0.15	-0.25	-0.71	-0.16	-0.35	0.16
	Lachnospiraceae	9	*Eubacterium rectale (100)*	-0.11	-0.08	-0.14	-0.09	-0.11	-0.13	0.02	0.00	0.02
		16	*butyrate-producing bacterium SS3/4 (100)*	-0.13	-0.07	-0.09	-0.10	-0.10	-0.18	-0.01	-0.05	-0.01
		18	*Fusicatenibacter saccharivorans (100)*	-0.02	-0.17	0.03	-0.18	-0.14	-0.21	**-0.14**	-0.10	-0.02
		22	*Ruminococcus faecis (100)*	0.05	0.18	-0.03	0.13	0.25	0.29	0.11	0.22	0.06
		27	*Corynebacterium sp. SN15 (100) , Blautia sp. GD8 (100)*	0.19	-0.12	-0.04	-0.14	0.13	-0.01	-0.18	0.16	0.13
		29	*Dorea longicatena (100)*	-0.05	-0.30	-0.03	-0.16	-0.20	-0.42	**-0.20**	-0.21	-0.08
		35	*Clostridium clostridioforme (100) / bolteae (100)*	-0.19	**-0.33**	-0.24	-0.52	-0.21	-0.60	-0.27	-0.02	0.12
		46	*Clostridium XlVa sp. (93)*	-0.07	-0.26	-0.09	**-0.41**	-0.15	**-0.68**	**-0.35**	-0.16	0.09
	Oscillospiraceae	52	*Oscillibacter sp. (78)*	0.20	0.20	-0.08	0.18	-0.06	-0.24	0.03	-0.20	0.15
	Ruminococcaceae	5	*Faecalibacterium prausnitzii (100)*	0.02	-0.06	0.05	0.08	0.09	-0.11	-0.08	-0.08	-0.09
		21	*Gemmiger formicilis (100)*	**0.19**	-0.04	**0.20**	-0.12	**0.15**	-0.29	**-0.33**	-0.11	0.04
		44	*Faecalibacterium prausnitzii (100)*	0.04	-0.21	-0.09	-0.15	-0.21	-0.51	-0.20	-0.24	0.04
		45	*Ruminococcus bromii (99)*	0.34	0.38	0.38	0.44	0.40	0.12	-0.06	-0.15	-0.04
	Selenomonadaceae	1	*Clostridiales bacterium Art 12/1 (98)*	0.30	0.34	0.28	0.28	0.30	0.36	0.03	0.05	0.04
	Veillonellaceae	12	*Dialister succinatiphilus (100)*	0.25	0.36	0.27	0.40	0.37	0.22	0.03	-0.04	-0.03
Proteobacteria	*Enterobacteriaceae*	3	*Escherichia coli (100) / vulneris (100) , Shigella sonnei (100) , Photorhabdus luminescens (100)*	-0.27	-0.17	-0.14	-0.04	-0.15	-0.31	0.01	-0.14	-0.13
	Sutterellaceae	36	*Unclassified Sutterellaceae (87)*	-0.12	0.04	-0.10	-0.03	-0.07	-0.08	0.07	-0.01	0.03
		51	*Parasutterella excrementihominis (97)*	0.15	0.01	0.18	-0.04	0.08	-0.55	**-0.33**	-0.30	0.01
		55	*Sutterella sp. 252 (98)*	-0.02	0.14	0.03	-0.10	0.01	0.00	0.00	0.04	0.09
Verrucomicrobiota	*Akkermansiaceae*	39	*Akkermansia sp. (94)*	**0.14**	0.18	0.07	0.07	-0.01	0.04	0.03	-0.05	0.10

Table S6. Average normalized microbial shifts and effects of the different treatment parameters (pasteurization, starting culture and type of water) on the most abundant OTUs detected after 48h of incubation. Positive values indicate enrichment by the treatment and are indicated in green. Statistical significance of an enrichment is indicated in bold ($p<0.05$). K1 = starting culture 1; K2 = starting culture 2; NP = non-pasteurized; P = pasteurized

Phylum	Family	OTU	Closely related species	Average normalized microbial shifts						Treatment effects		
				K1 H		K1 V		K2 V		P-NP	K2-K1	HB-VB
				NP	P	NP	P	NP	P			
Actinobacteria	*Bifidobacteriaceae*	2	*Bifidobacterium adolescentis (100) / faecale (100)*	0.24	0.42	0.05	0.31	0.35	0.75	**0.28**	**0.37**	**0.15**
		11	*Bifidobacterium longum subsp. null (100) / longum subsp. longum (100)*	0.19	0.35	0.05	**0.29**	0.31	**0.37**	0.15	0.17	0.10
		30	*Bifidobacterium longum subsp. Null (98) / longum subsp. Longum (98)*	0.27	0.34	0.06	**0.29**	0.35	**0.44**	0.13	**0.22**	0.13
		37	*Bifidobacterium pseudocatenulatum (100) / kashiwanohense (100) / catenulatum (100)*	0.44	0.51	0.43	0.57	0.59	0.86	0.15	**0.22**	-0.03
	Coriobacteriaceae	8	*Collinsella aerofaciens (100)*	-0.14	-0.45	-0.17	-0.49	-0.64	0.22	0.08	0.13	0.04
	Eggerthellaceae	43	*Coriobacteriaceae bacterium WAL 18889 (98), Senegalimassilia anaerobia (97)*	0.13	0.04	0.03	-0.02	0.00	0.13	0.00	0.06	0.08
Bacteroidetes	*Bacteroidaceae*	4	*Bacteroides vulgatus (100)*	-0.25	-0.59	-0.10	-0.50	-0.57	-0.14	-0.10	-0.05	-0.12
		6	*Bacteroides uniformis (100)*	0.02	-0.05	0.03	-0.08	-0.16	0.10	0.03	0.00	0.02
		7	*Bacteroides uniformis (100)*	0.06	-0.07	0.06	-0.12	-0.22	0.07	0.00	-0.04	0.03
		13	*Bacteroides eggerthii (100)*	0.15	0.13	0.10	-0.02	0.04	0.44	0.09	0.20	**0.10**
		15	*Bacteroides stercoris (100)*	0.04	-0.10	-0.01	0.28	-0.18	0.12	0.15	-0.16	-0.16
		19	*Bacteroides plebeius (100)*	0.00	0.25	-0.20	0.03	0.13	0.37	0.24	0.34	0.21
		23	*Bacteroides faecis (100) / thetaiotaomicron (100)*	-0.19	-0.05	0.03	-0.13	-0.19	-0.43	-0.09	**-0.26**	-0.07
		31	*Bacteroides thetaiotaomicron (100)*	0.00	-0.13	0.05	**-0.15**	0.02	-0.07	-0.14	0.02	-0.02
		33	*Bacteroides ovatus (100)*	**0.04**	-0.04	-0.03	0.01	0.10	0.15	0.00	0.13	0.01
		34	*Bacteroides fragilis (100)*	0.21	0.12	0.18	0.02	0.01	0.06	-0.07	-0.07	0.06
		40	*Bacteroides massiliensis (100)*	-0.24	-0.61	-0.37	-0.31	-0.76	-0.49	-0.01	-0.29	-0.09
		42	*Bacteroides sp. (82)*	0.91	0.40	0.61	0.13	0.52	0.38	**-0.38**	0.08	**0.28**
		48	*Bacteroides sp. (77)*	0.28	-0.09	0.11	-0.21	-0.15	-0.15	**-0.23**	-0.10	0.14
		49	*Bacteroides coprophilus (97)*	0.21	0.06	0.07	0.16	0.00	0.21	0.05	0.00	0.02
	Porphyromonadaceae	38	*Unclassified Porphyromonadaceae (62)*	0.20	0.00	0.15	-0.12	-0.02	-0.31	-0.25	-0.18	0.08
	Prevotellaceae	14	*Prevotella sp. (68)*	0.38	0.42	0.14	0.48	0.33	0.26	0.10	-0.02	0.08
		41	*Prevotella sp. (96)*	0.16	-0.20	0.12	-0.04	0.08	0.64	0.01	0.32	-0.06
	Rikenellaceae	17	*Alistipes sp. (85)*	0.27	0.19	0.23	0.04	0.11	-0.07	-0.15	-0.11	0.09
		24	*Alistipes finegoldii (100) / onderdonkii (100)*	0.20	0.01	0.12	-0.01	-0.13	-0.23	-0.14	-0.23	0.05
		26	*Alistipes putredinis (100)*	-0.02	-0.09	-0.04	**-0.21**	-0.19	**-0.17**	-0.07	-0.06	0.06
	Tannerellaceae	20	*Parabacteroides distasonis (99)*	-0.05	-0.10	-0.01	-0.10	-0.12	-0.15	-0.06	-0.08	-0.03
		28	*Bacteroides merdae (100) , Parabacteroides sp. S448 (100)*	0.17	0.13	0.07	0.35	0.01	0.16	0.13	-0.12	-0.06
Desulfobacterota	*Desulfovibrionaceae*	62	*Desulfovibrio sp. (96)*	0.22	0.21	0.26	0.20	0.28	0.66	0.10	0.24	-0.01
Firmicutes	*Acidaminococcaceae*	10	*Clostridium carnis (100) , Phascolarctobacterium faecium (100)*	0.10	-0.11	0.06	0.07	-0.08	-0.18	-0.10	-0.20	-0.07
	Christensenellaceae	32	*Unclassified Clostridiales (81)*	0.19	-0.03	0.11	-0.08	-0.08	-0.01	-0.11	-0.06	0.07
	Erysipelotrichaceae	25	*Holdemanella sp. (91)*	-0.33	-0.66	-0.24	-0.38	-0.16	-0.19	-0.16	0.13	-0.19
	Lachnospiraceae	9	*Eubacterium rectale (100)*	-0.07	0.00	-0.23	-0.10	-0.04	0.43	**0.23**	0.36	0.13
		16	*butyrate-producing bacterium SS3/4 (100)*	**-0.17**	-0.08	-0.09	-0.10	0.04	0.05	0.03	0.14	-0.03
		18	*Fusicatenibacter saccharivorans (100)*	-0.05	-0.10	0.03	-0.20	-0.17	0.00	-0.04	0.00	0.01
		22	*Ruminococcus faecis (100)*	-0.03	0.27	-0.12	0.22	0.17	0.53	**0.34**	0.30	0.07
		27	*Corynebacterium sp. SN15 (100) , Blautia sp. GD8 (100)*	**0.33**	0.34	0.13	0.12	0.51	0.37	-0.05	0.32	0.21
		29	*Dorea longicatena (100)*	**-0.16**	-0.17	**-0.03**	-0.25	-0.32	-0.23	-0.05	-0.13	-0.02
		35	*Clostridium clostridioforme (100) / bolteae (100)*	-0.20	-0.20	-0.38	-0.69	-0.46	0.08	0.08	0.34	0.33
		46	*Clostridium XlVa sp. (93)*	0.18	0.08	-0.02	-0.05	-0.19	-0.14	-0.03	-0.13	0.17
	Oscillospiraceae	52	*Oscillibacter sp. (78)*	0.04	-0.04	**-0.22**	-0.27	**-0.11**	-0.52	-0.18	-0.07	0.24
	Ruminococcaceae	5	*Faecalibacterium prausnitzii (100)*	0.11	0.13	0.20	0.13	0.16	0.10	-0.03	-0.03	-0.04
		21	*Gemmiger formicilis (100)*	0.12	-0.06	0.03	**-0.22**	-0.07	**-0.08**	-0.15	0.02	0.12
		44	*Faecalibacterium prausnitzii (100)*	**0.14**	0.07	**0.09**	0.02	**-0.10**	-0.17	-0.07	-0.19	0.04
		45	*Ruminococcus bromii (99)*	0.35	0.16	0.32	0.18	0.22	0.24	-0.11	-0.02	0.01
	Selenomonadaceae	1	*Clostridiales bacterium Art 12/1 (98)*	0.40	0.37	0.32	0.33	0.35	0.42	0.02	0.06	0.06
	Veillonellaceae	12	*Dialister succinatiphilus (100)*	0.39	0.21	0.31	0.17	0.23	0.36	-0.06	0.05	0.06
Proteobacteria	*Enterobacteriaceae*	3	*Escherichia coli (100) / vulneris (100) , Shigella sonnei (100) , Photorhabdus luminescens (100)*	-0.17	-0.28	-0.22	-0.31	-0.29	-0.58	-0.17	-0.17	0.04
	Sutterellaceae	36	*Unclassified Sutterellaceae (87)*	0.01	-0.12	0.00	0.10	-0.13	-0.01	0.03	-0.12	-0.10
		51	*Parasutterella excrementihominis (97)*	0.28	0.07	0.19	-0.10	-0.11	-0.15	-0.18	-0.18	0.13
		55	*Sutterella sp. 252 (98)*	0.16	0.20	0.18	0.17	0.07	0.49	0.15	0.10	0.00
Verrucomicrobiota	*Akkermansiaceae*	39	*Akkermansia sp. (94)*	0.00	-0.10	-0.04	**-0.10**	-0.06	0.11	0.00	0.09	0.02

Supplementary Figures

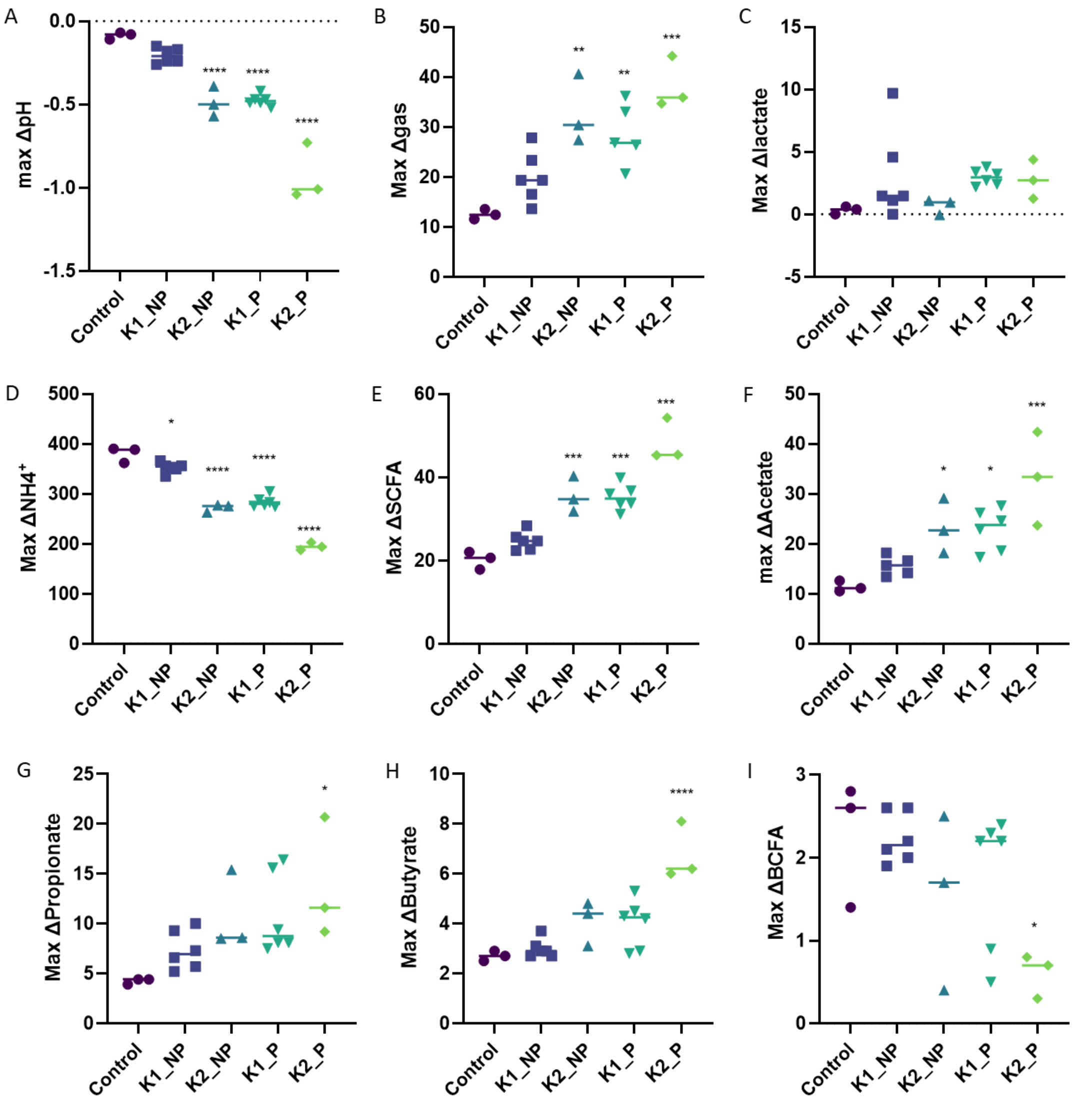

Figure S1. Short-term effect of different kefir products on microbial activity of healthy donors. Graphs represent the maximum variation (Δ) respect to time 0 for different fermentation markers pH (A), gas (B), lactate (C), NH4+ (D), total SCFA (E), acetate (F), propionate (G), butyrate (H), BCFA (I) (n = 3 donors). Significant differences compared to the control are represented by (*), (**), (***) and (****) representing p<0.05, p<0.01, p<0.001 and p<0.0001, respectively.

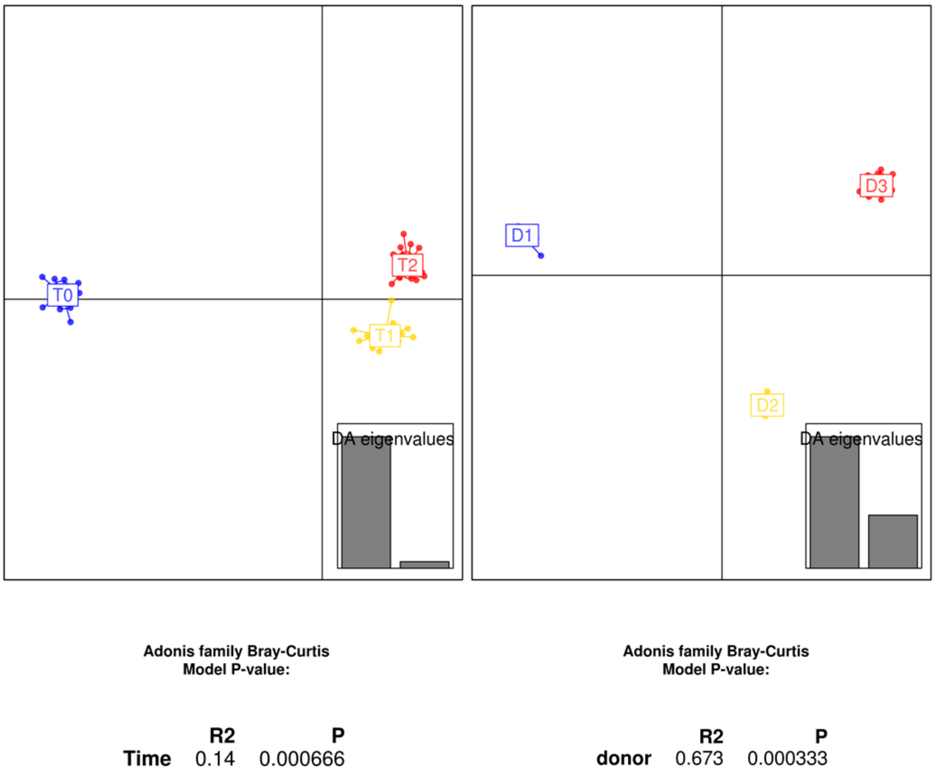

Figure S2. Discriminant Analysis of Principal Components and Adonis based on Bray-Curtis distance for time and donor at family level. T0 = start of the experiment, T1 = 24h, T2 = 48h. D = donor.

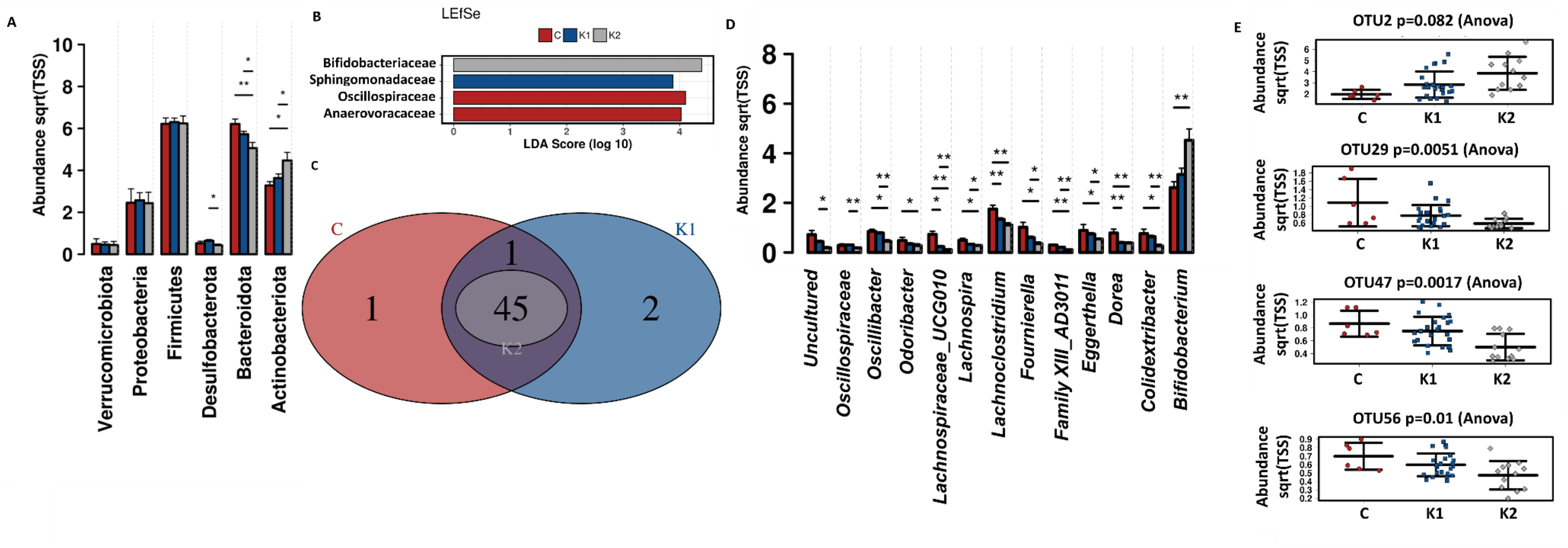

Figure S3. Effect of different starting cultures of water kefir on microbial structure in vitro. (A) Bar plot of the abundance at phylum level. Statistically significant differences between control and water kefir condition are marked by asterisks [p<0.05 (*), p<0.01 (**),p<0.001 (***)]. (B) Linear discriminant effect size (LEfSe) analysis at family level. (C) Venn diagram of shared and unique features between control and kefir treatments based on abundance and occurrence data. (D) Bar plot of the abundance at genus level of statistically significant [p<0.05 (*), p<0.01 (**),p<0.001 (***)] differences between control and kefir condition. (E) Diversity indices Shannon, Chao1 and Simpson's index.

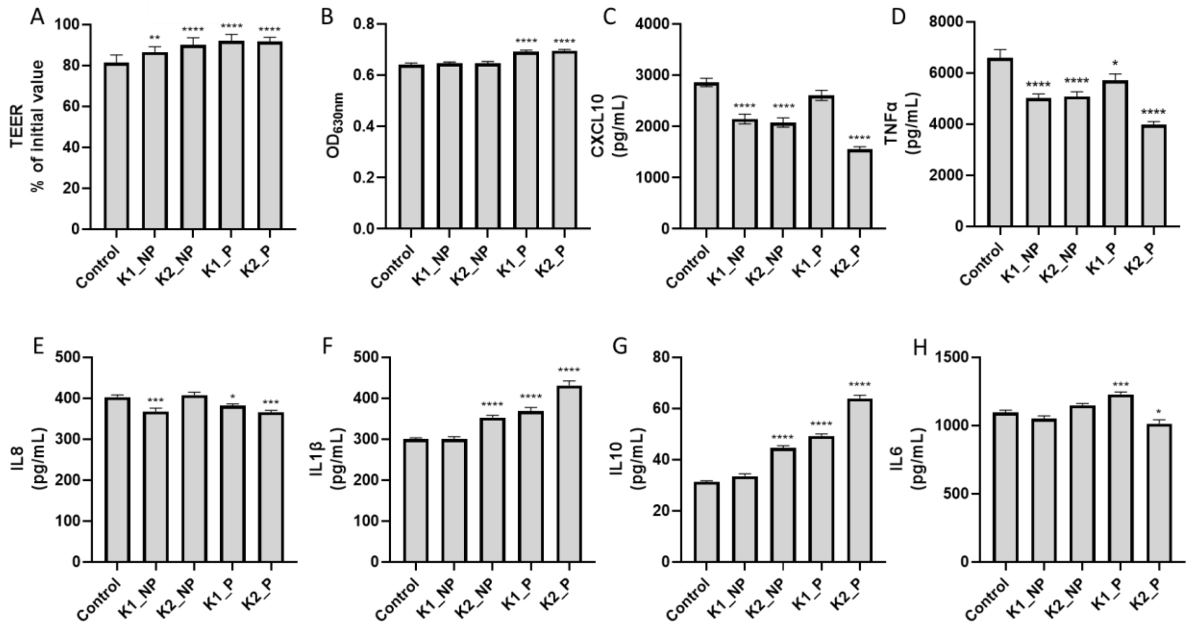

Figure S4. Effect of different products on epithelial barrier and immune modulation. Bars represent the mean ± SEM (n ≥ 3) for TEER percentages of the initial value (A), NFκβ activity of THP1 cells (B), CXCL10 (C), TNFα (D), IL8 (E), IL1β (F), IL10 (G) and IL6 (H) in the basolateral media. Significant differences between control condition are marked by (*), (**) and (***)p<0.05, p<0.01 and p<0.001, respectively.

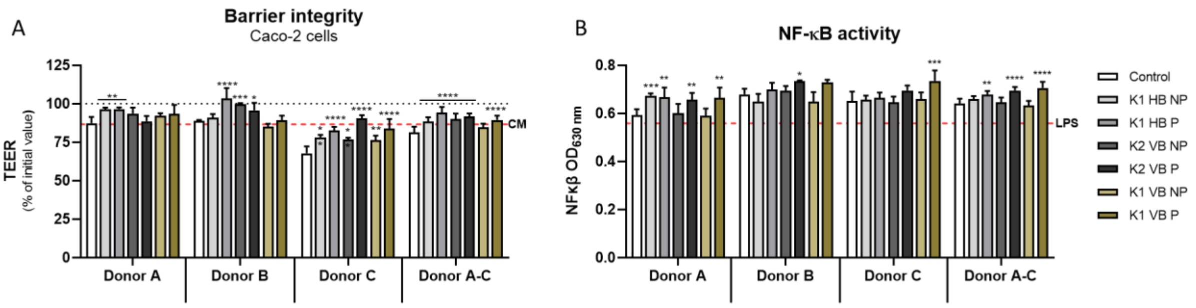

Figure S5. Effect of colonic batch suspensions on transepithelial electrical resistance (TEER) of the Caco-2/THP1-BlueTM co-cultures (A) and NFκβ activity of THP1-Blue™ cells (B), including interindividual differences. TEER was measured 24h after pretreatment of the co-cultures and each 24h value was normalized to its corresponding 0h value and is shown as percentage of initial value. The grey dotted line represents 100% (initial value). The red dotted line corresponds to the experimental control CM (complete medium). NFκβ activity levels were measured 6h after LPS treatment on the basolateral side of the Caco-2/THP1-Blue™ co-cultures after pre-treatment of the apical side for 24h with the colonic batch suspensions. The red dotted line corresponds to the experimental control LPS+. Data are plotted as mean ± SEM. (*) represents statistically significant differences between the control and treatment samples. (*) = p<0.05; (**) = p<0.01; (***) = p<0.001; (****) = p<0.0001. NP = non-pasteurized; P = pasteurized; Donor A-C = average of all 3 donors

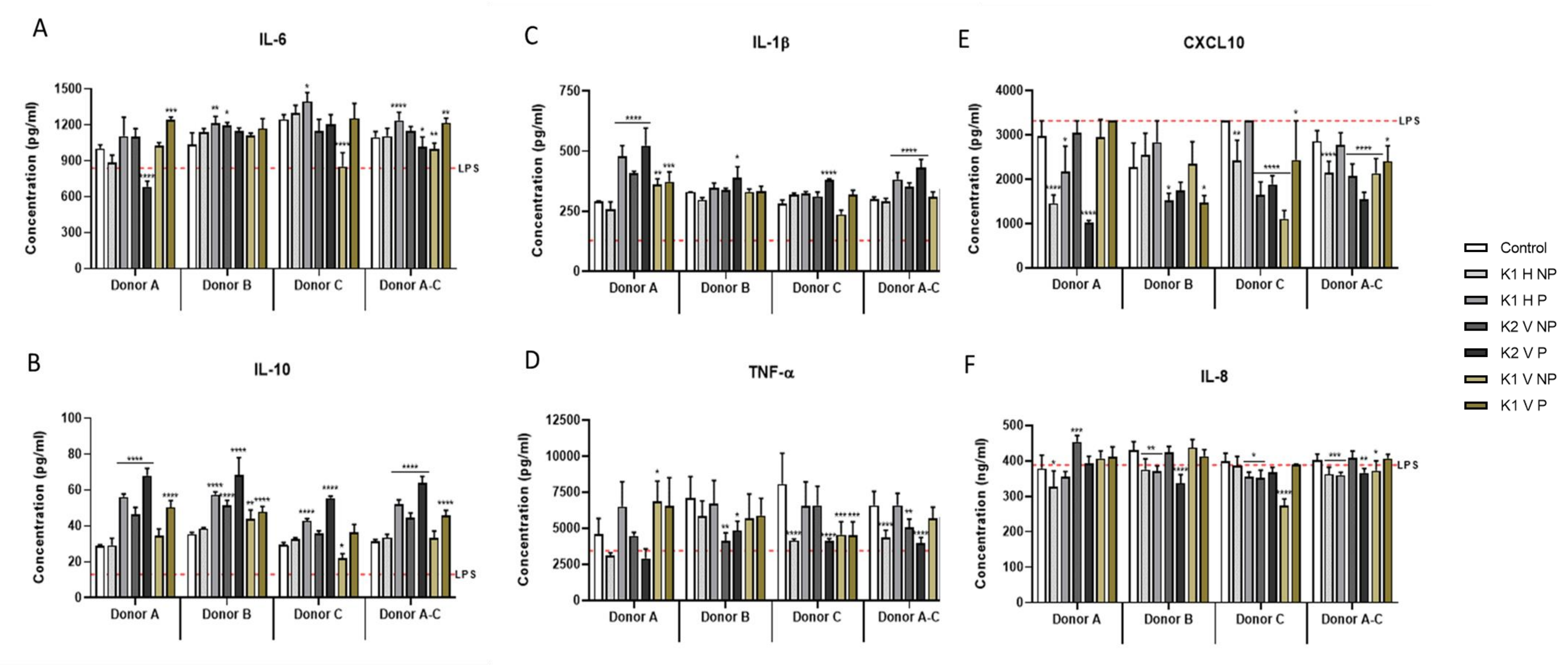

Figure S6: Effect of colonic batch suspensions on secretion of IL-6 (A), IL-10 (B), IL-1β (C), TNF-α (D), CXCL10 (E) and IL-8 (F) including interindividual differences. Cytokine levels were measured 6h after LPS treatment on the basolateral side of the Caco-2/THP1-Blue™ co-cultures after pre-treatment of the apical side for 24h with colonic batch suspensions. The red dotted line corresponds to the experimental control LPS+. Data are plotted as mean ± SEM. (*) represents statistically significant differences between the control and treatment samples. (*) = p<0.05; (**) = p<0.01; (***) = p<0.001; (****) = p<0.0001.; NP = non-pasteurized; P = pasteurized; Donor A-C = average of all 3 donors
